# Size-Dependent Thresholds in CuO Nanowires: Investigation of Growth from Microstructured Thin Films for Gas Sensing

**DOI:** 10.3390/nano14141207

**Published:** 2024-07-16

**Authors:** Christian Maier, Verena Leitgeb, Larissa Egger, Anton Köck

**Affiliations:** 1 Materials Center Leoben Forschung GmbH, Roseggerstrasse 12, 8700 Leoben, Austria; verena.leitgeb@mcl.at (V.L.); larissa.egger@mcl.at (L.E.); 2 Institute for Chemistry and Technology of Materials, TU Graz, Stremayrgasse 9, 8010 Graz, Austria

**Keywords:** nanowires, microstructures, copper oxide, thin films, gas sensing

## Abstract

An experimental characterization of cupric oxide nanowire (CuO NW) growth from thermally oxidized, microstructured Cu thin films is performed. We have systematically studied the influence of the thickness and dimension of Cu layers on the synthesis of CuO NW. The objective was to determine the optimum Cu geometries for increased CuO NWs growth to bridge the gap between adjacent Cu structures directly on the chip for gas sensing applications. Thresholds for CuO-NW growth regarding film thickness and lateral dimensions are identified based on SEM images. For a film thickness of 560 nm, NWs with lengths > 500 nm start to grow from the edges of Cu structures with an area ≥ 4 µm^2^. NWs growing from the upper surface were observed for an area ≥ 16 µm^2^. NW growth between adjacent thermally oxidized thin films was analyzed. The study provides information on the most relevant parameters of CuO NWs growth, which is mandatory for integrating CuO NWs as gas sensor components directly on microchips. Based on this result, the gap size of the structure was varied to find the optimum value of 3 µm.

## 1. Introduction

Metal oxide sensors are one promising candidate due to having a fast reaction speed, high response, low production cost, and high integration possibility [1]. In general, metal oxide sensors can be subdivided into potentiometric, amperometric, and resistive types based on their measurement principle [2]. The interest in resistive gas sensors is a result of their easy fabrication and high stability. The sensing mechanism is based on an interaction of gas molecules with the sensitive layer. The reaction with gaseous molecules causes a change in the resistance by the formation or reduction of charge carriers such as holes or electrons [3]. One-dimensional nanostructures provide superior long-term stability due to their high crystallinity and lack of grain boundaries [4,5]. The main advantage of using nanostructured materials for gas sensors is a high surface to volume ratio, which means that the surface properties are becoming more important. An electrical change on the surface has a very high influence on the electrical resistance of the nanomaterials, which means that the sensor has high sensitivity [6]. In times of high energy prices, the need for more efficient sensors with less power consumption is increasing [7].

Nanowire integration with standard CMOS technology is particularly interesting, as it may lead to increased functionality and superior performance in combination with low-cost batch fabrication. There are many different metal oxide gas sensors based on nanowires such as ZnO, SnO_2_, TiO_2_, WO_3,_ or CuO [8]. Ahn et al. showed a highly sensitive nanowire gas sensor based on a bridging of nanowires between two electrodes [9]. There are many published results that demonstrate the high importance and possibilities of CuO-nanowires for the detection of different gasses like CO [10], H_2_S [11], NOx [8], H_2_ [12], and VOCs [13]. In recent years, CuO NWs have been the subject of investigation for their potential use in the removal of a wide range of volatile organic compounds (VOCs), including methanol, acetonitrile, isopropanol, acetone, ethanol, and toluene [14,15].

The development of nanowires for sensing applications is strongly increasing. Bottom-up and top-down methods are the two approaches for nanowire integration in CMOS technology [16,17]. The bottom-up method is the dominating synthesis approach, due to the main advantage that the nanowires can be fabricated on any substrate [16]. Bottom-up synthesis can be carried out in the solution or vapor phase. The first strategy contains nanowire synthesis and a transfer step to the substrate. There are different techniques like drop casting, Langmuir–Blodgett, blow-bubble, contact printing, or spray coating to deposit the nanowires onto a substrate. In contrast, the vapor phase methods are based on the vapor–liquid–solid mechanism and includes methods such as chemical vapor deposition [18].

The technology of choice for realizing a NW-based chemical sensors would be the on-chip fabrication of NWs in order to avoid such complicated and usually expensive techniques. A feasible approach for the integration of CuO nanowires as sensing devices without any post-processing steps was demonstrated in [19]. Steinhauer et al. employed CuO nanowires synthesized directly on-chip by thermal oxidation of electroplated copper structures with a thickness of 2.5 µm. The CuO NWs bridge the gap between copper structures and form a “multi NW grid” structure, which has the desired high surface to volume ratio for gas sensing. In this case, the CuO-NWs forming the sensor components are fully suspended and surrounded by ambient air, which provides an optimum sensor configuration. This specific thermal oxidation process is cheap, occurs without a catalyst, and is possible at temperatures (<400 °C) compatible with standard CMOS technology, which is essential for integration inro standardized industrial microelectronic process technology. It is also reported in the literature that thermal oxidation is feasible directly on MEMS-based resistive microheaters, which makes heating the whole sensor in a furnace unnecessary [20].

As shown in [21,22], the main mechanism of CuO nanowire synthesis is based on the diffusion of Cu cations via grain boundaries. Cu cations pass the large, columnar Cu_2_O layer and the nanocrystalline CuO layer that is formed upon oxidation of the Cu structure. Then, the Cu cations diffuse at the CuO surface and along the nanowire sidewalls towards the tip. In general, there are many complex aspects that need to be considered, including position, growth direction, synthesis temperature, morphology, and crystal structure, for successful nanowire device implementation. In the literature, NW growth is frequently studied on thick Cu foils with dimensions of 20 × 5 mm^2^ [23], where oxidation temperatures exceeding 400 °C [24] are employed, which are not feasible to utilize on micro-hotplate chips. Additionally, studies [25] have been conducted on Si substrates with Cu thicknesses of 1000 and 1500 nm, which are not applicable for small structures.

The research on materials such as CuO is of high interest, due to the fact that CuO enables bridging the gaps between adjacent thermally oxidized structures by multiple nanowires. The main advantage of such bridging technology is that no additional nanowire transfer process and no further fabrication steps are required after nanowire synthesis [8,26]. Moreover, this approach avoids any liquid organic chemistry, as used for photo- and e-beam lithographic steps, which might contaminate the CuO-NW surface and is detrimental for sensor performance.

The objective of this study is to deepen the experimental knowledge for fabricating a CuO-NW sensor on fully CMOS-integrated micro-hotplate chips [27]. The main focus is set on the determination of the optimum parameters (gap size, thickness, length, and width of the Cu structures) for enhanced NW growth directly on the 80 × 80 µm^2^ sized micro-hotplate. Hence, the size of the Cu structures and the process parameters (e.g., thermal oxidation temperature < 400 °C) are strictly limited due to the micro-hotplate size, and the CMOS integration. The parameter study was performed on Si substrates with a thin SiO_2_ layer on the top substrate; later, the best parameter was tested on a sensor chip.

## 2. Materials and Methods

CuO NWs were synthesized by thermal oxidation of microstructured Cu thin films with different thicknesses and sizes. A thin Cr layer was used as an intermediate layer between the substrate and the Cu thin films, to reduce Cu diffusion into the substrate during the thermal oxidation step and to improve thin film adhesion. For the following investigations, the films were deposited on Si substrates with a thin SiO_2_ layer on top.

### 2.1. Fabrication of Microstructured Cu Thin Films

Microstructured Cu thin films were fabricated by electron beam lithography (EBL) using the positive resist polymethyl methacrylate (PMMA) on the Si substrates. The EBL system Raith eLINE+, Raith GmbH (Dortmund, Germany), was used. After electron exposure and chemical resist development, Cr and Cu thin films were subsequently evaporated onto the resulting PMMA mask. Cr and Cu layers were deposited by thermal evaporation (UNIVEX 450, Leybold GmbH, Cologne, Germany), with a deposition rate of 0.07 ± 0.01 nm/s for Cr and 0.5 ± 0.1 nm/s for Cu at 4.7 × 10^−6^ mbar. The thickness of the adhesion layer was 15 nm Cr; the Cu film thicknesses were 415 ± 5 nm, 590 ± 5 nm, 750 ± 5 nm, and 945 ± 5 nm.

After deposition, a lift-off in acetone followed and the layer thickness was measured by AFM.

**Figure 1:**
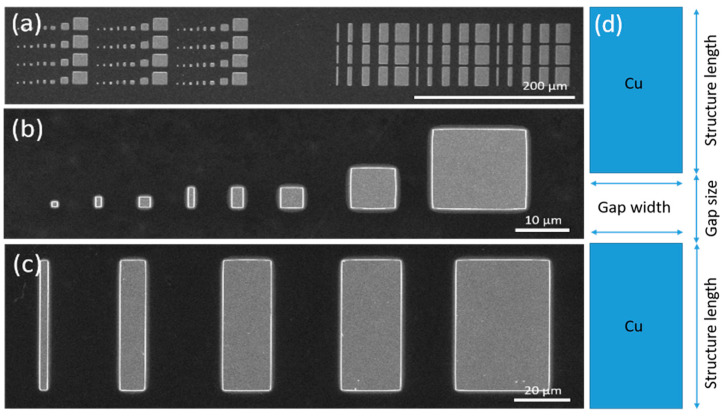
SEM images of Cu microstructures fabricated by EBL. (**a**) Overview of the structures ‘Squares-variation’ (**left**) and ‘Gap-variation (i)’ (**right**) and (**b**,**c**) zoomed-in SEM images. (**d**) Designation of structures.

**Figure 2:**
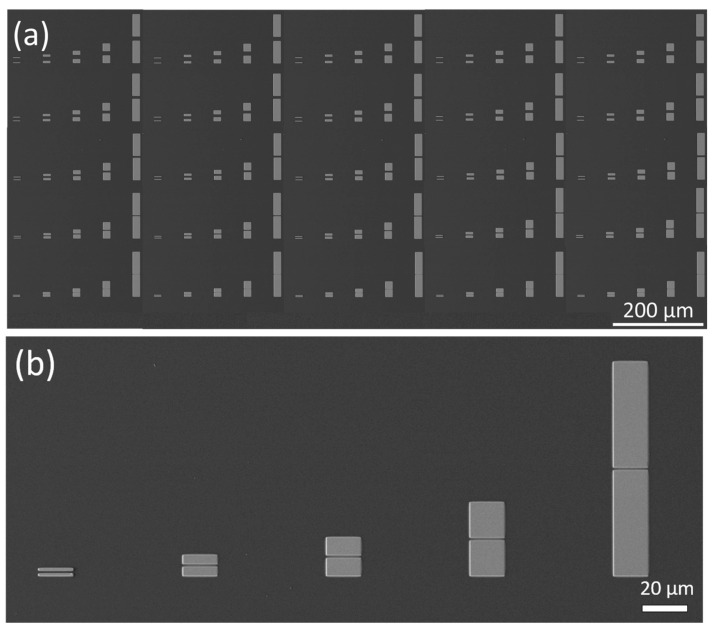
SEM images of Cu microstructures fabricated by EBL. (**a**) Overview of the structures ‘Gap-variation (ii)’ and (**b**) zoomed in SEM image on one variation in length (field).

In the following, two different structures are chosen for the systematic investigation and are denoted as (i) ‘Squares-variation’ and (i–ii) ‘Gap-variation’. Figure 1 and Figure 2 shows a SEM image of both Cu structures before thermal oxidation.

‘Squares-variation’: Figure 1a,b consists of 12 identical fields, each field consisting of 8 Cu squares with dimensions 1 × 1 µm^2^, 1 × 2 µm^2^, 2 × 2 µm^2^, 1 × 4 µm^2^, 2 × 4 µm^2^, 4 × 4 µm^2^, 8 × 8 µm^2^, and 16 × 16 µm^2^, with a thickness of the Cu layer of 560 ± 5 nm.

‘Gap-variation’ included several samples: a structure consisting of two adjacent rectangles was varied with respect to (i) gap width (Figure 1a,c) and (ii) structure length and gap size (Figure 2a,b). On the structures of (ii), the layer thickness was further varied. As shown in Figure 1c ‘Gap-variation (i)’ consists of 9 identical fields with 25 µm long rectangles of increasing width (1 µm, 4 µm, 8 µm, 10 µm, and 16 µm) and constant gaps of 5 µm between their short sides. The layer thickness was 560 ± 5 nm.

Structure ‘Gap-variation (ii)’, see Figure 2, consisted of 5 identical fields with rectangles of constant gap width (16 µm) From left to right, the structure length was varied (1 µm, 4 µm, 8 µm, 16 µm, and 46 µm). In an upward direction, the gap size was varied (1.5 µm, 3 µm, 5 µm, 7 µm, and 10 µm). These structures were fabricated 5 times, in order to investigate the repeatability with the following layer thicknesses (Cr/Cu): 415 ± 5 nm, 590 ± 5 nm, 750 ± 5 nm, and 945 ± 5 nm. Length specification in the following always refers to these structure sizes before thermal oxidation.

### 2.2. Synthesis of CuO NW

The Cu thin films were thermally oxidized for 4 h at 355 ± 5 °C, using a PZ28-3T hotplate (HP, Harry Gestigkeit GmbH, Düsseldorf, Germany) in ambient air and pressure (1 bar). The temperature was controlled using a thermocouple. Samples were put on the HP at room temperature (25 °C). The temperature was increased to 355 °C over a period of approximately 15 min, resulting in a heating rate of 22 °C per minute. After a holding time of 4 h, the HP was turned off. A cooling rate of 2 °C was employed, resulting in a cooling period of approximately 3 h before the samples were removed from the HP. All samples were treated with citric acid monohydrate (0.1 mol/L mixture, with distilled water) directly before the oxidation process to remove any oxide layer from the sample surface. Oxidation parameters were chosen based on experiences from previous works [28]. The primitive cell of CuO has four atoms, which leads to 12 vibrations modes, that are expressed with Equation (1). *A_g_* + *B_g_* are the Raman-active modes. *A_u_* + *B_u_* are infrared-active.

(1) $$\Gamma_{CuO}=A_{g}+2B_{g}+4A_{u}+5B_{u}$$

Equation (2) shows the modes of Cu_2_O. In contrast to CuO, Cu_2_O has only the *T*_2*g*_ mode, which is Raman-active. The *A*_2*u*_ + *E_u_* are infrared active and the *T*_1*u*_ + *T*_2*u*_ are silent.

(2) $$\Gamma_{Cu_{2}O}=A_{2u}+E_{u}+3T_{1u}+T_{2u}+T_{2g}$$

**Figure 3:**
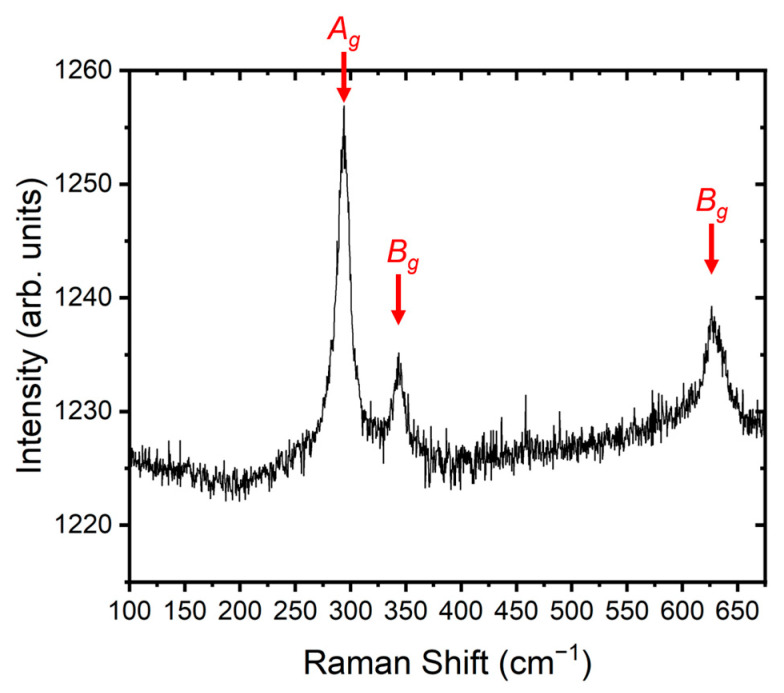
Raman investigation on the CuO structures.

Measurement was performed with a Raman spectrometer (WITec alpha 300, WITec GmbH, Ulm, Germany) with a Zeiss EC Epiplan-Neofluar DIC 100×/0.9 NA objective (Carl Zeiss Microscopy GmbH, Oberkochen, Germany). The spectral range that was used covers the range between 100 and 700 cm^−1^ (1800 g/mm grating). In Figure 3, all Raman active modes (A_g_: 294; B_g_: 345 and 627) of CuO were clearly identified and also correlates with the literature [29,30]. Also, no Cu_2_O phases were detected with the measurement.

## 3. Results

### 3.1. Size-Dependent Thresholds for NW Growth from Microstructured Cu Thin Films

The growth of CuO NWs on thermally oxidized, microstructured Cu thin films was investigated to identify the threshold for NW growth depending on the lateral dimensions of the thin films.

**Figure 4:**
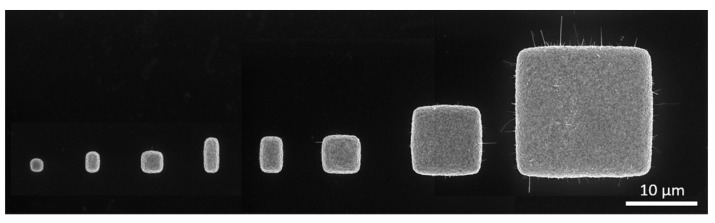
SEM image showing CuO NWs synthesized by thermal oxidation of Cu microstructures of increasing lateral dimensions (1 × 1 µm^2^, 1 × 2 µm^2^, 2 × 2 µm^2^, 1 × 4 µm^2^, 2 × 4 µm^2^, 4 × 4 µm^2^, 8 × 8 µm^2^, and 16 × 16 µm^2^); film thickness: 560 nm.

Figure 4 shows a SEM image of one exemplary field of the structure ‘Squares-variation’, illustrating the observed increase in growth of CuO NWs with increasing lateral dimensions. The number of CuO NWs per square was counted on SEM images of squares, see Table 1.

**Figure 5:**
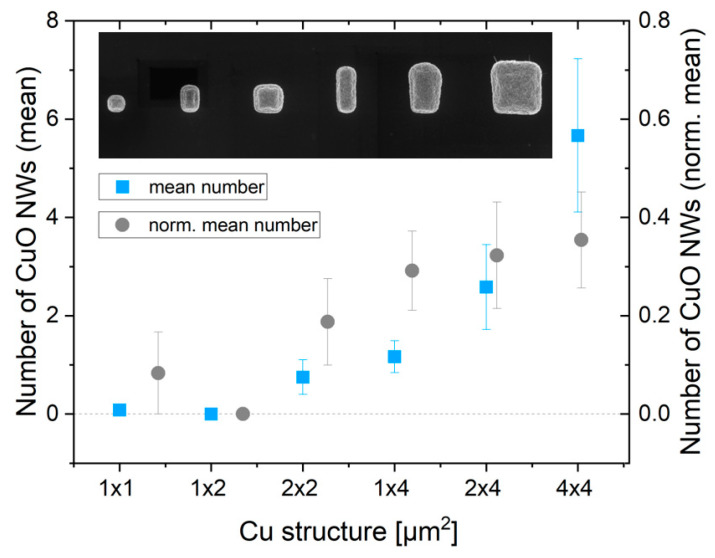
Mean number of CuO NWs synthesized by thermal oxidation of Cu microstructures, dependent on their lateral dimension (blue squares) and mean values normalized to 1 × 1 µm^2^ unit area (gray circles).

For each square size, the mean value of CuO NWs was taken over 12 squares of the same dimensions. For comparison of NW growth on squares of different dimensions, the mean values were normalized to 1 × 1 µm^2^ unit area, as shown in Figure 5.

NW growth started for squares with an area larger than 2 µm^2^, with one exception on field 9, where a CuO NW about 100 nm long grew from the edge of an 1 × 1 µm^2^ square. With increasing area, the total number of NW increased. Regarding the normalized number of NW, a saturation was observed around 8 µm^2^. Growth of long CuO NWs (>500 nm) was only observed for squares with areas ≥ 4 µm^2^.

**Figure 6:**
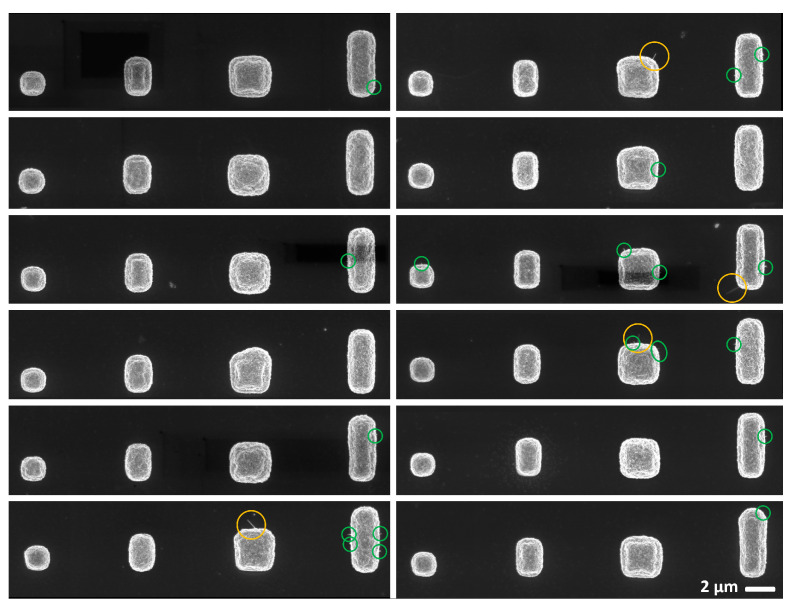
SEM images of the four smallest squares (1 × 1 µm^2^, 1 × 2 µm^2^, 2 × 2 µm^2^, and 1 × 4 µm^2^) of fields 1–12 of the structure ‘Squares-variation’. Yellow circles mark CuO NW > 500 nm and green circles mark NW stumps growing from the edges of the structures.

In Figure 6, SEM images illustrate the dependence of NW growth on the structure size. The growth of CuO NWs started at the edges of the structures, which was to be expected due to the concentration of structural stress/strain near the free edges [28,31].

No growth of NWs was observed on the upper surface of the first four squares with areas < 2 × 4 µm^2^. Furthermore, NWs reached a maximum length of 500 nm on squares with areas < 4 × 4 µm^2^.

Comparing NW growth on square and rectangular structures (Table 2) with the same areas showed a slightly higher mean number of NWs for the rectangular structures. A possible explanation could be the higher stress within structures of a higher aspect ratio, which leads to an increase in NW growth.

On the other hand, counting only NWs of lengths > 500 nm, more NWs were observed on the 2 × 2 µm^2^ squares (3/12 squares) than on the 1 × 4 µm^2^ squares (1/12 squares). Here, the lower aspect ratio could be favorable for increased diffusion of Cu cations towards the growing NWs, leading to the increased length.

### 3.2. CuO NWs Bridging Gaps

**Figure 7:**
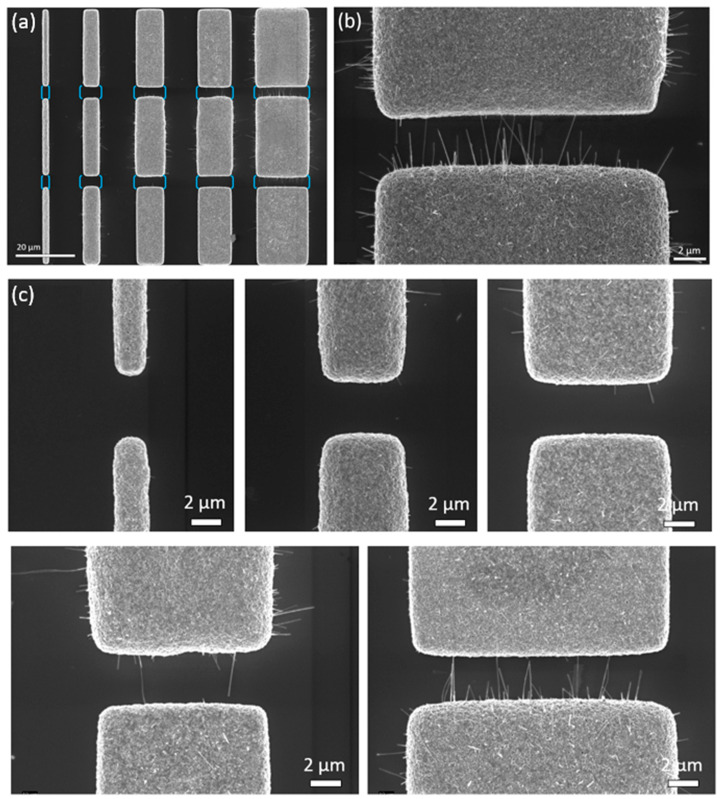
CuO NWs bridging the gaps between oxidized Cu rectangles of 25 µm structure length and varied gap width. (**a**) SEM image of fields 1 to 3 of the structure ‘Gap-variation (i)’; blue brackets mark gaps where CuO NW growth was analyzed. (**b**) Zoomed-in view of gap between the 16 × 25 µm^2^ rectangles of fields 1 and 2. (**c**) Zoomed-in view of the gaps between the rectangles of fields 2 and 3 (width 1 µm, 4 µm, 8 µm, 10 µm, and 16 µm).

With respect to the realization of chemical sensors, the dependence of the growth behavior of CuO NWs bridging gaps across adjacent structures on the dimension of the microstructured Cu films was investigated. Therefore, the structures of ‘Gap-variation’ were properly designed with a constant gap size of 5 µm. For the first ‘Gap-variation (i)’, the CuO NWs’ growth behavior across the gaps between rectangles of different gap width was analyzed, as shown in Figure 7.

The Cu rectangles were fabricated with a 5 µm gap size between their short sides. During the thermal oxidation process, the initial gap size of 5 µm between rectangles was reduced due to the “swelling” of the two copper oxide layers (Cu_2_O and CuO) on the Cu surface. The gap size was reduced to about 4 µm (1 µm gap width), 3.5 µm (4 µm gap width), and 3 µm (8, 10, and 16 µm gap width), respectively.

**Figure 8:**
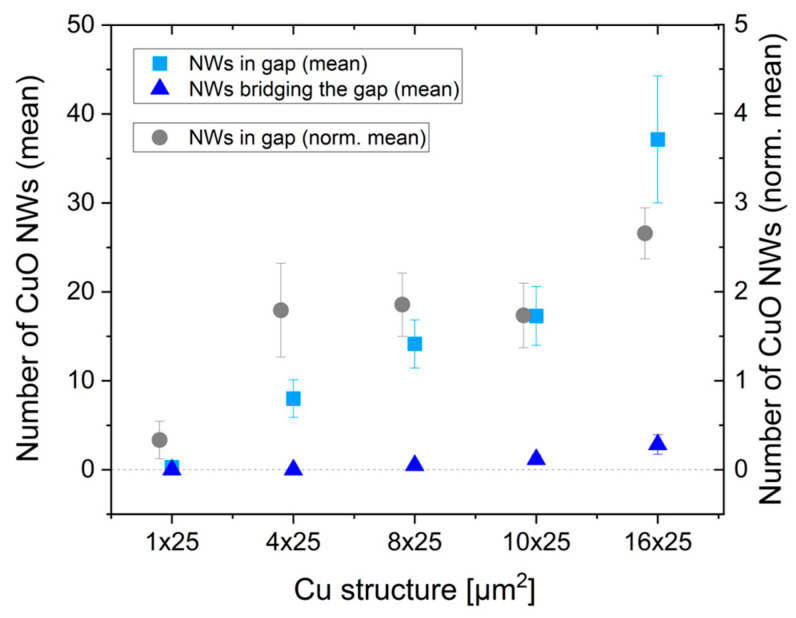
Mean number of CuO NWs growing across the gap (blue squares) and bridging the gap (dark blue triangles) between the short sides of oxidized Cu rectangles, dependent on their lateral dimensions and mean values normalized to the width of the oxidized Cu rectangles (gray circles).

Table 3a,b, together with Figure 8, give an overview of the CuO NW growths in the gaps, dependent on the structure width of the Cu rectangles. With increasing structure width, the mean number of CuO NWs in the gap increased from 0.3 ± 0.2 (1 µm structure width) to 37.1 ± 7.1 (16 µm structure width). Normalizing the number of CuO NWs to the width showed an increase from about 0 to about 2 CuO NWs per unit length when increasing the width from 1 µm to 4 µm. The normalized number stayed constant for further increases to 8 µm and 10 µm width and increased to about 2.7 CuO NWs per unit length for width 16 µm.

For sensing applications, the number of CuO NWs bridging the gap is important. For structure width 1 µm and 4 µm, no CuO NWs bridging the gap were found. Single CuO NWs long enough to cross the gap were found for 8 µm width (on three-sixths of gaps). For widths of 10 µm and 16 µm, the mean number of NWs bridging the gap was about 1.2 (four-sixths of gaps) and 2.8 NWs (five-sixths of gaps) per gap, respectively (corresponding to about 0.1 and 0.2 NW per unit length, respectively). The fact that no bridging CuO NWs were found for the gaps between the 4 µm wide rectangles could also be due to the larger gap size of 3.5 µm after oxidation, as compared to the 3 µm gap size between the bigger rectangles.

**Figure 9:**
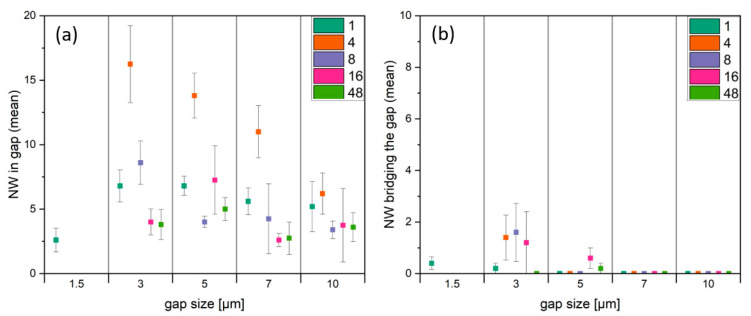
Mean numbers of CuO NWs (**a**) in the gaps and (**b**) bridging the gaps of 16 µm wide rectangles with different lengths, as given in the inset, dependent on the gap size.

Based on the demonstrated increase in NW growth for structures with larger widths, the structure ‘Gap-variation (ii)’ was designed with rectangles of 16 µm structure width. The influence of the variation in gap size and length of Cu rectangles on NW growth across the gaps was investigated. The Cu film thickness was 590 ± 5 nm. Figure 9 shows the dependence of NW growth on the gap size for structures with different lengths. All NWs in the gap were counted, which includes both the ‘bridging’ and the ‘non-bridging’ NWs.

For structures with a length larger than 1 µm, the gaps with size of 1.5 µm are closed (Figure 9a), caused by the swelling of the Cu structures during the oxidation process. For all other gap sizes, NW growth inside the gap occurred. The highest number of NWs was identified on a structure length of 4 µm (Figure 9, marked in orange). The gap size seemed to have no influence on the NW growth.

**Figure 10:**
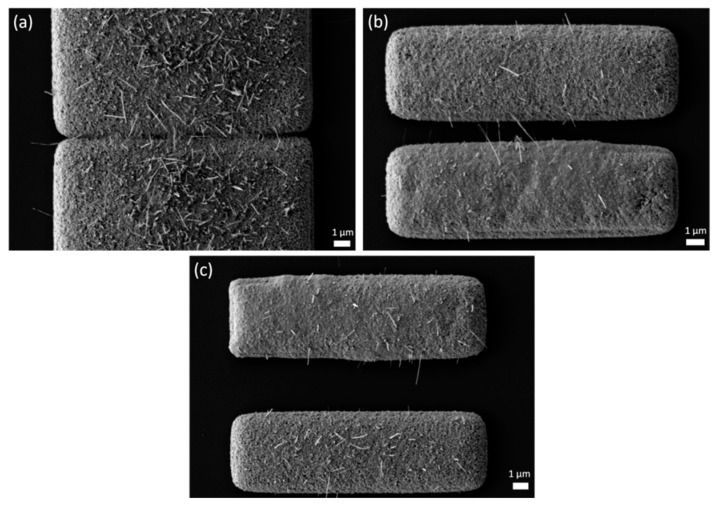
SEM images of CuO microstructures with a film thickness of 590 ± 5 nm. (**a**) Gap with 1.5 µm size closed after oxidation. (**b**) Gap with 10 µm size with no NWs bridging. (**c**) Gap with 3 µm size with bridging NWs.

However, the gap size played an important role considering the bridging NWs. The mean numbers of CuO NWs bridging the gaps, dependent of the gap size and the structure length, are shown in Figure 9b. A gap size of 3 µm (Figure 10b) caused the most bridging NWs. Increasing the gap size from 3 µm to 5 µm halved the number of bridging NW. As mentioned before, the gap size of 1.5 µm led to a closing of the gaps for structure lengths > 1 µm. No bridging NWs were observed on the 7 and 10 µm gap structures, due to the long distance (Figure 10c). Also, a structure length of 1 µm causes NW growth but no bridging of the gap. This can be explained as the amount of copper was not sufficient for NW to grow long enough.

### 3.3. Thickness-Dependent NW Growth from Cu Thin Films

**Figure 11:**
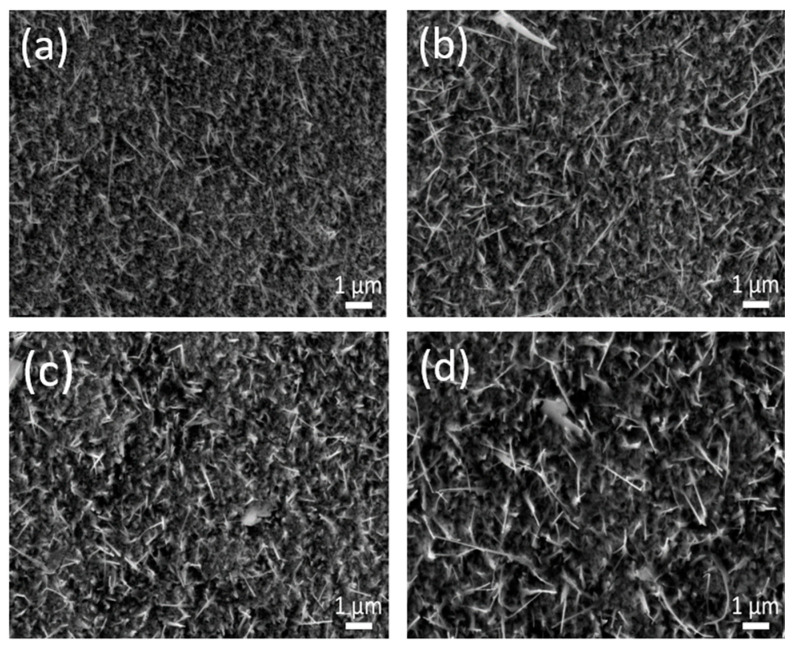
SEM image of CuO NWs synthesized by thermal oxidation at 355 °C and 4 h of Cu thin films with thicknesses (**a**) 415 nm, (**b**) 590 nm, (**c**) 750 nm, and (**d**) 945 nm ± 5 nm.

The dependence of NW growth on Cu film thickness was also investigated by employing Cu films with different layer thickness (415, 590, 755, and 945 ± 5 nm). Figure 11 shows the SEM images of these Cu thin films after thermal oxidation. For all thicknesses, NW growth was observed. With increasing layer thickness, the density and diameters of the CuO NWs increased, which is in accordance with Ref. [32].

**Figure 12:**
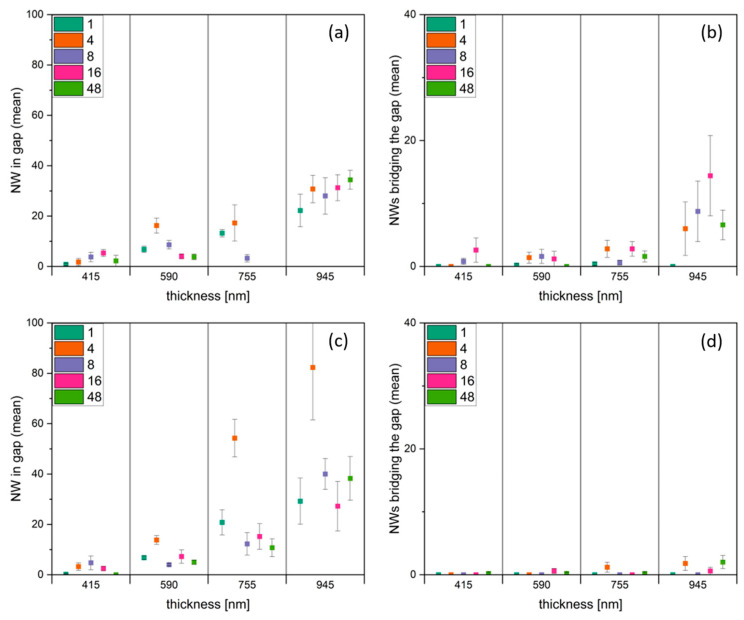
Mean numbers of CuO NWs (**a**,**c**) in the gap and (**b**,**d**) bridging the gap, dependent on CuO thickness for structure lengths, as given in the inset. Gap sizes (**a**,**b**) 3 µm and (**c**,**d**) 5 µm.

In the next step, the number of NWs in the gaps were investigated for different film thicknesses and gap sizes. As shown in Figure 12, the number of NWs grown in the gap increases with increasing film thickness. Figure 12 shows the results for 3 µm and 5 µm gap size. Gap size 1.5 µm was not considered due to the mentioned gap closing. For gap sizes 7 µm and 10 µm, no bridging of NWs was observed for all layer thicknesses. Increasing the film thickness from 415 nm to 945 nm led to an about 4 to 5 times higher number of NWs growing in the gap. For all investigated thicknesses, the strongest NW growth was observed again on the 4 µm long structures (Figure 12a,c marked in orange).

Considering NWs bridging the gap (Figure 12b,d), for the 3 µm gap, the number increased 7 times when increasing the film thickness from 417 nm to 945 nm (example taken from the 16 µm long structure). For 750 nm film thickness and structures of 1 µm length, bridging NWs were observed on the 3 µm gap. For the 5 µm gap, bridging started at higher film thicknesses than for the 3 µm gap, with less bridging NWs for all film thicknesses.

## 4. Realization of Chemical Sensors

For a highly sensitive, chemo-resistive-based gas sensor, a large specific surface area is a key factor. Therefore, a high number of NWs connecting both Cu structures is desirable. In order to fabricate Cu gas sensor with a high number of NWs, we chose the best parameters from the previous results. The fabricated gas sensor has a targeted CuO gap size of 3 µm. The oxidation parameters, derived from the preceding outcomes, were utilized. To enable electrical contacts to the oxidized Cu structures, which are bridged by the CuO-NWs, the Cu structures were fabricated on specific Si platform chips with integrated Pt-electrodes. However, the length of the Cu rectangle had to be modified to match with the geometry of the Pt electrodes on the platform chip.

**Figure 13:**
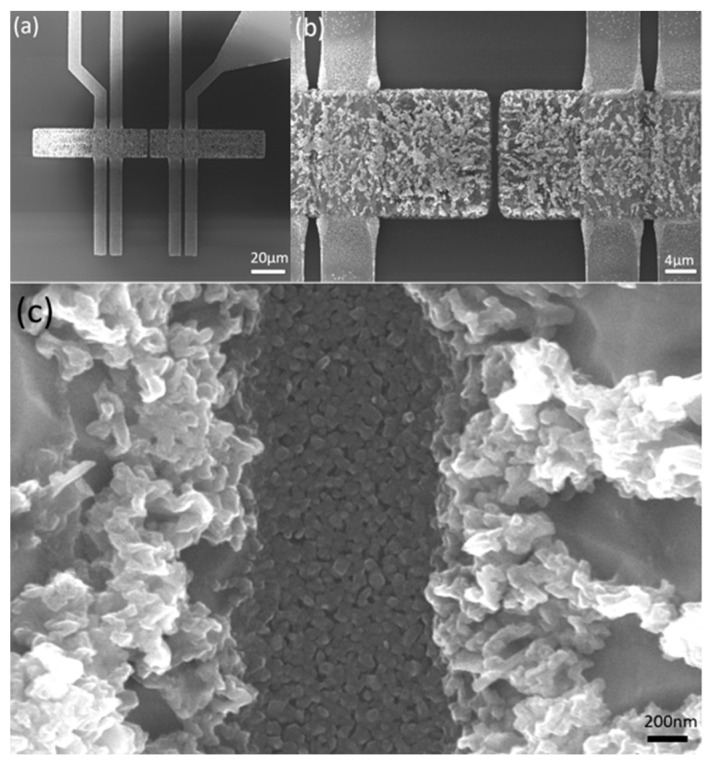
SEM images of the fabricated gas sensor: (**a**) overview of the sensor structure, (**b**) diffusion along the Pt electrodes, and (**c**) growing of the structure in the gap.

The fabrication of CuO NW structures on the platform chip by thermal oxidation introduced new technological challenges. The oxidation of the Cu caused a significant diffusion of Cu material along the Pt electrode (Figure 13a,b), which obviously reduced the stress in the substrate and prevented efficient CuO-NW growth. In order to compensate for this effect, the gap width was increased up to 32 µm and the length of the rectangle was increased up to 70.5 µm (Figure 13a). Also, the Cr adhesion layer thickness was increased to 25 nm. Besides the diffusion along the Pt electrodes, the swelling of the Cu structures is another stress-releasing effect, which prevents CuO-NW growth. Instead, the CuO structures grow together at the bottom of the gap (Figure 13c), which provides an electrical connection and enables operation as a sensor device. The thickness of the Cu was 590 ± 5 nm, due to the fact that higher thicknesses delaminated on the sensor platform chip.

A specific gas measurement setup was used to characterize the sensing performance. Determination of the resistance was conducted by measuring the voltage in a controlled atmosphere with synthetic air (Linde Gas GmbH, Dublin, Ireland) consisting of a composition of 80% nitrogen and 20% oxygen. A constant flow of 1000 sccm was set by a mass flow controller (EL-FLOW, Bronkhorst High-Tech B.V, Ruurlo, The Netherlands). The relative humidity (r.h.) was achieved by the gas flow through a bubbler system and was checked by a commercial humidity sensor (AFK-E, KOBOLD Holding Gesellschaft m.b.H., Vienna, Austria). Here, the relative humidity was set to 50%. For a preliminary performance test, the sensor was operated at a temperature of 300 °C and exposed to CO_2_ with 1000, 2000, and 4000 ppm. Furthermore, the sensor was evaluated against CO concentrations of 5, 10, and 20 ppm, as well as a specific HCmix test gas (equal mixture of 500 ppm of acetylene, ethane, ethene, and propene). The test gas concentration was chosen based on typical indoor air quality limits. The change in resistance was caused by reactions with the target gas. The sensor responses (*S*) are calculated after Equation (3), where *R_g_* is the resistance during gas exposure and *R_a_* is the resistance in synthetic air.

(3) $$S=\frac{R_{g}-R_{a}}{R_{a}}\times 100\%$$

**Figure 14:**
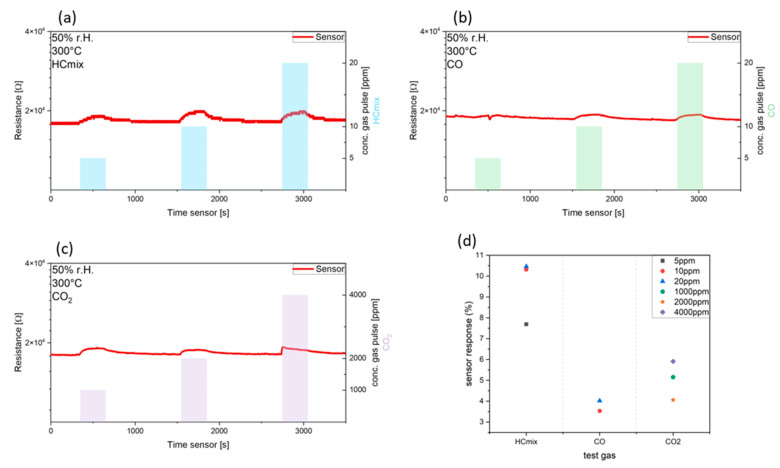
Resistance measurement of the fabricated CuO sensor against 5, 10, and 20 ppm exposure of HCmix (**a**) or CO (**b**) and 1000, 2000, and 4000 ppm for CO_2_ (**c**) at an operating temperature of 300 °C and 50% r.h.; (**d**) Summarized sensor responses.

The resistance measurement and the sensor response to the test gasses are illustrated in Figure 14. The sensor exhibits a response of 7.7% for 5 ppm of HCmix (Figure 14a), reaching saturation at 10 and 20 ppm (10.3 and 10.4%, respectively). In the case of CO exposure, the sensor did not exhibit a response at 5 ppm. For 10 and 20 ppm of CO (Figure 14b), a sensor response of 3.5% and 4% was achieved. The sensor also demonstrated a response to CO_2_ (Figure 14c) for 1000, 2000, and 4000 ppm with 5.1%, 4.1%, and 5.9%. Here, a saturation was also observed, as shown in Figure 14d, which summarizes the sensor responses for the different concentrations.

**Figure 15:**
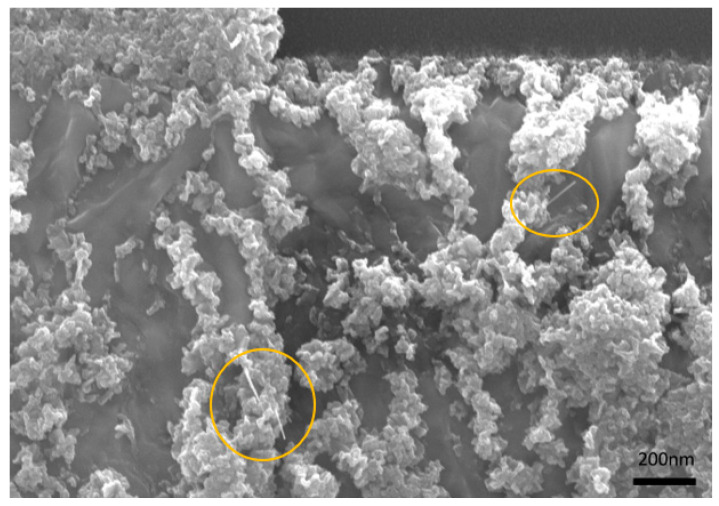
SEM images of the fabricated gas sensor with NWs (yellow circles) on the surface of the CuO structures.

Nevertheless, no NW growth was achieved in the gap, but some NWs were observed on the surface of the structures and are indicated in Figure 15.

## 5. Summary and Outlook

In this work, we have systematically studied the influence of the thickness and dimension of Cu layers on the synthesis of CuO-NW by thermal oxidation in order to determine the optimum Cu geometries for efficient NW growth on Si substrates. This study has been performed to realize chemical sensors employing CuO-NWs, which bridge and electrically connect two adjacent CuO structures. We have found that the growth of CuO-NWs (Cu film thickness of 560 nm) is initiated if the structure has an area larger than 4 µm^2^; the number of CuO-NWs further increases with the area. The optimum parameters for CuO-NWs bridging the 3 and 5 µm gap sizes were investigated, the best result was obtained for structures of 4 × 16 µm^2^ size for all investigated Cu film thicknesses.

Based on this result, the gap size of the structure was changed to find the optimum value. For gap sizes smaller than 3 µm, the gaps closed during the thermal oxidation process of CuO due to swelling of the whole structures. Gap sizes larger than 5 µm cannot be bridged by the CuO-NWs. The gap size of 3 µm resulted in the highest number of bridging NWs, which seems to be the optimum for realizing CuO-NW sensors.

However, although we have found the essential parameters for optimum CuO-NW growth on Si substrates, the experimental results cannot be directly transferred to practical sensor configurations, which require electrical contacts to the oxidized Cu structures. We have used specific Si platform chips with integrated Pt electrodes for implementation of the Cu structures, which has a strong impact on NW growth behavior. Diffusion of the Cu along the Pt electrodes results in a reduction in stress in the Cu materials during thermal oxidation. To increase the stress in the material, the gap width was increased to 32 µm and the Cr adhesion layer thickness was increased to 25 nm. However, in this case, no CuO-NW growth across the gaps was achieved. Instead, the CuO-film grew together at the bottom of the gap and provided sensor operation. Thus, we were able to test this sensor using the test gas HCmix. Nevertheless, the sensor response for 10 and 20 ppm at 10.3 and 10.4% is not high and shows saturation for higher concentrations. In addition, other test gasses, such as CO and CO_2_, also demonstrate low responses of 4 and 5.9%, respectively.

In order to fully exploit the findings of this study for miniaturized chemical nanosensor devices employing suspended CuO-NWs as sensor components—an optimum sensor configuration—we will develop more sophisticated electrode configurations, which have no impact on CuO-NW growth. This might be achieved by fabricating bridging CuO-NW structures, as a first step, followed by an additional processing step (photolithograpy, metal evaporation, and lift-off) to electrically contact the adjacent CuO-NW bridged structures. However, this process might result in breakage of the delicate CuO-NWs. Thus, our approach will be to use additional barrier materials (e.g., TiN) between the Pt electrodes and the Cu structures to prevent the diffusion of the Cu material. Also, the use of different geometries—area of underlying TiN-diffusion barrier layer larger than the Cu structure—might prevent the Cu from diffusing along the Pt electrodes. This work is presently under progress.

## Figures and Tables

**Table 1:** Number of CuO NWs synthesized by thermal oxidation of microstructured Cu thin films of 560 nm thickness; the lateral dimensions are as given in the table. Mean values highlighted gray (bottom row) are normalized to 1 × 1 µm^2^ unit area. The “field number” indicates the 12× repeated structures, as shown in Figure 1a.

	Number of CuO NWs					
Field	1 × 1 µm^2^	1 × 2 µm^2^	2 × 2 µm^2^	1 × 4 µm^2^	2 × 4 µm^2^	4 × 4 µm^2^
1	0	0	0	1	0	6
2	0	0	0	0	0	4
3	0	0	0	1	4	19
4	0	0	0	0	0	1
5	0	0	0	1	0	6
6	0	0	1	4	6	12
7	0	0	1	2	1	9
8	0	0	1	0	0	1
9	1	0	2	2	2	2
10	0	0	4	1	3	4
11	0	0	0	1	8	1
12	0	0	0	1	7	3
mean	0.1 ± 0.1	0 ± 0	0.8 ± 0.3	1.2 ± 0.3	2.6 ± 0.9	5.7 ± 1.6
norm. mean	0.1 ± 0.1	0 ± 0	0.2 ± 0.1	0.3 ± 0.1	0.3 ± 0.1	0.4 ± 0.1

**Table 2:** NW growth on square and rectangular microstructed, thermally oxidized CuO films (560 nm).

	Number of NWs (NWs Top + Side)			
Structure	2 × 2 µm^2^	1 × 4 µm^2^	4 × 4 µm^2^	1 × 16 µm^2^
1-1	0	1 (0 + 1)	6 (5 + 1)	10 (0 + 10)
1-2	0	0	4 (4 + 0)	2 (0 + 2)
1-3	0	1 (0 + 1)	19 (12 + 7)	2 (1 + 1)
1-4	0	0	1 (0 + 1)	-
2-1	0	1 (0 + 1)	6 (5 + 1)	19 (0 + 19)
2-2	1 (1 + 0)	4 (0 + 4)	12 (7 + 5)	6 (0 + 6)
2-3	1 (1 + 0)	2 (0 + 2)	9 (0 + 0)	6 (0 + 6)
2-4	1 (0 + 1)	0	1 (0 + 1)	-
3-1	2 (1 + 1)	2 (1 + 1)	3 (3 + 0)	6 (0 + 6)
3-2	4 (2 + 2)	1 (0 + 1)	4 (2 + 2)	9 (1 + 8)
3-3	0	1 (0 + 1)	1 (1 + 0)	12 (0 + 12)
3-4	0	1 (1 + 0)	3 (2 + 1)	-
mean	0.8 (0.4 + 0.3)	1.2 (0.2 + 1)	5.8 (3.4 + 1.6)	8 (0.2 + 7.8)

**Table 3:** (**a**) Number of CuO NWs in gaps and NWs bridging the gap between the short sides of 25 µm long oxidized Cu rectangles; the width is as given in the table head. The field numbers indicate the investigated gaps shown in Figure 7a. (**b**). Number of CuO NWs normalized to width of oxidized Cu rectangle.

	**(a) Number of NWs in Gap (NWs Bridging Gap)**				
structure width	1 µm	4 µm	8 µm	10 µm	16 µm
field 1–2	0	2	5	17 (1)	39 (5)
field 2–3	0	4	18	21 (3)	57 (7)
field 3–4	1	4	11	3	46 (1)
field 4–5	0	7	25 (1)	28 (2)	23
field 6–7	0	10	18 (1)	23 (1)	46 (1)
field 8–9	1	16	12 (1)	12	44 (3)
mean	0.3 ± 0.2	8 ± 2	14 ± 3	17 ± 3	37 ± 7
	**(b) Number of NWs in Gap Normalized to Width**				
structure width	1 µm	4 µm	8 µm	10 µm	16 µm
field 1–2	0	0.5	0.6	1.7	2.4
field 2–3	0	1	2.3	2.1	3.6
field 3–4	1	1	1.4	0.3	2.9
field 4–5	0	1.8	3.1	2.8	1.4
field 6–7	0	2.5	2.3	2.3	2.9
field 8–9	1	4	1.5	1.2	2.75
norm. mean	0.3 ± 0.2	1.8 ± 0.5	1.9 ± 0.4	1.7 ± 0.4	2.7 ± 0.3

## Data Availability

Data are contained within the article.
